# Subtotal Petrosectomy (SP) in Cochlear Implantation (CI): A Report of 92 Cases

**DOI:** 10.3390/audiolres12020014

**Published:** 2022-02-25

**Authors:** Ignacio Arístegui, Gracia Aranguez, José Carlos Casqueiro, Manuel Gutiérrez-Triguero, Almudena del Pozo, Miguel Arístegui

**Affiliations:** 1 Otoneurology Division and Otology Section, ENT Department, Hospital General Universitario Gregorio Marañón, 28007 Madrid, Spain; ignaris11@gmail.com; 2 Pediatric ENT Section, ENT Department, Hospital General Universitario Gregorio Marañón, 28007 Madrid, Spain; gracia.aranguez@salud.madrid.org; 3 Otology Section, ENT Department, Hospital Severo Ochoa, 28007 Madrid, Spain; jccasqueiro@gmail.com; 4 Otology Section, ENT Department, Hospital General Universitario Gregorio Marañón, 28007 Madrid, Spain; guram64@yahoo.es (M.G.-T.); a_delpozo@yahho.es (A.d.P.); 5 ENT Department, Hospital General Universitario Gregorio Marañón, 28007 Madrid, Spain

**Keywords:** cochlear implantation, subtotal petrosectomy, complications in cochlear implantation, vestibular schwannoma, complex cases in cochlear implantation, inner ear malformations, Ménière’s disease, chronic otitis media, petrous bone cholesteatoma, temporal bone fracture

## Abstract

In most cases, cochlear implantation is a straightforward procedure. Nevertheless, there are clinical situations in which the presence of the middle ear may compromise access and/or the outcome in terms of complications. This article includes a series of patients for whom we eliminated the middle ear to facilitate placement of the electrode array of the implant and/or reduce potential complications. A total of 92 cases in 83 patients, managed by the senior author, are included in this series. Different indications are outlined that justify associating a subtotal petrosectomy technique with cochlear implantation. The steps of the technique are described. We include complications from this series that compare favorably with standard techniques.

## 1. Introduction

There are many pathological and clinical conditions in which the presence of the middle ear may pose a threatening condition, and eliminating it may help to definitively solve the problem, reducing risks for the patient. Chronic ear disease, petrous bone cholesteatoma, and meningoencephalic herniation are among these conditions [1]. The technique of subtotal petrosectomy (SP), associated with cochlear implantation (CI), was described to overcome clinical challenging conditions [2,3]. With the aim of providing protection against infection and extrusion of a cochlear implant, SP was proposed for many complex clinical cases [4,5,6,7,8,9,10]. Furthermore, when the anatomy is contracted, or access to the inner ear is compromised, eliminating the middle ear will provide the best promontorial access for electrode insertion. This article reviews a series of patients in which SP was associated with cochlear implantation. The technique, the conditions, the indications, and the outcome, in terms of complications, are shown and compared with the current literature. 

## 2. Materials and Methods

A retrospective review of patients operated on for cochlear implantation (CI) by the senior author, in which a subtotal petrosectomy was included in the procedure, was performed. Age, sex, indications, and postoperative complications were included. Patients were divided into different groups based on their pathological conditions.

In this series, implants from different companies were used (Cochlear©, Sidney, Australia; Medel©, Innsbruck, Austria; Advance Bionics©, Los Angeles, CA, USA; Digisonic©, Houston, TX, USA; Oticon©, Egedal, Denmark). 

### 2.1. Technique

**Figure 1:**
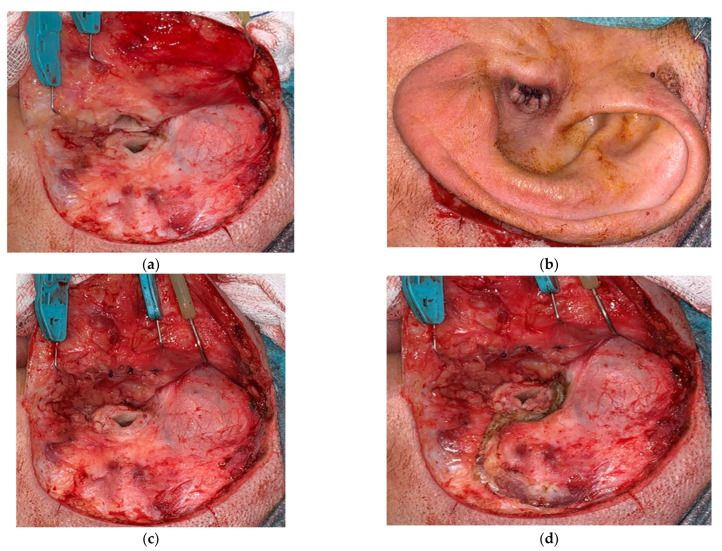

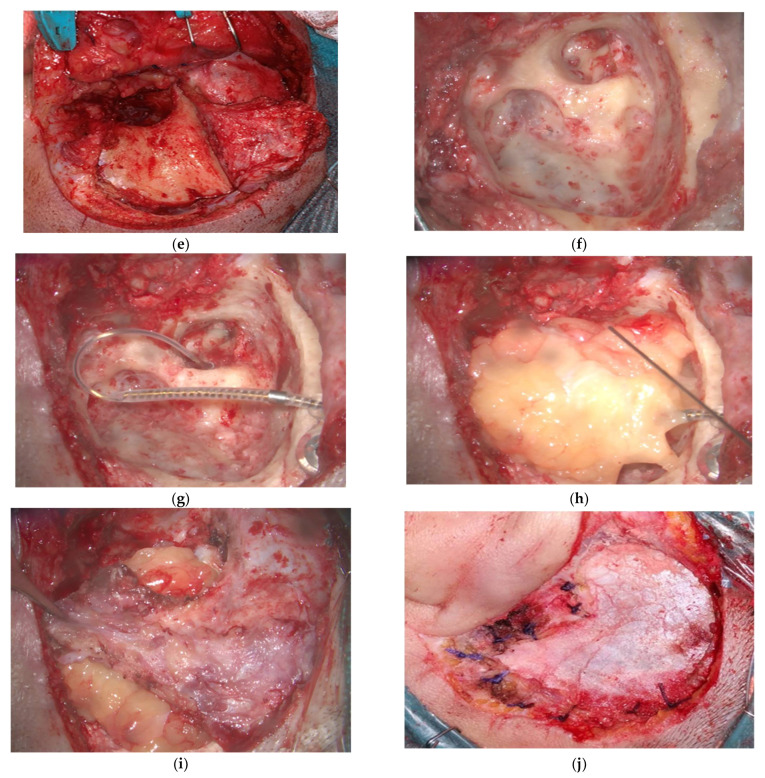
Subtotal petrosectomy. (**a**) Wide retroauricular incision with 360° sectioning of EAC. (**b**) Blind sac closure of EAC. (**c**) Second layer closure with tragal cartilage. (**d**) Musculoperiosteal incision U-shaped pedicle superiorly. (**e**) Musculoperiosteal flap reflected superiorly. (**f**) Elimination of temporal bone cells as required. (**g**) Placement of the implant and insertion of electrode array. (**h**) Fat is used to obliterate the cavity. (**i**) Musculoperiosteal flap repositioned. (**j**) Tight closure of periosteal flap.

A wide retroauricular incision was used in these cases. The external auditory canal (EAC) was transected 360° (Figure 1a), the skin was elevated from the tragal and conchal cartilage, everted, and sutured (blind sac closure of the EAC) (Figure 1b). The tragal cartilage was used as a second layer and sutured (Figure 1c). The musculoperiosteal layer was designed in a U-shaped fashion, pedicled superiorly, to cover and protect the area lodging the implant (Figure 1d,e). The transmastoid approach should eliminate all middle ear and mastoid cells, as well as perilabyrinthine, pericarotid, and apical cells as far as possible (Figure 1f). Depending on the case, other techniques were employed, such as labyrinthectomy or translabyrinthine access to the internal auditory canal (IAC). The Eustachian tube was closed using periostium or muscle, firmly packed after deep electrocoagulation of the mucosa up to the isthmus. The internal device of the implant was placed and stabilized, and the electrode array was inserted through the round window (RW) or a cochleostomy, depending on the case (Figure 1g). Fat is used to obliterate the cavity (Figure 1h) and a tight closure performed in layers (Figure 1i,j). Only rarely has cochlear implantation been staged. 

### 2.2. Patients Were Subjected to SP Based on Different Conditions That Make the Technique Worth Considering

Previous infection inside the middle ear increases the risk of infection after cochlear implantation and, thus, the risk of extrusion. Conditions like chronic otitis media, cholesteatoma, or even relapsing acute otitis media are among them. 

#### 2.2.1. Actual or Potential Cerebrospinal Fluid (CSF) Liquorrhea

The risk of meningitis when there is a known or potential CSF leakage compromises the future of the implant and the life of the patient, and it may require revision surgery. Even in a normal middle ear, the chance of a middle ear infection or contamination exists. Conditions such as spontaneous or unavoidable CSF leak are included here. 

#### 2.2.2. Concomitant Extradural or Intradural Tumors

Whenever there is a need for tumor removal through a transmastoid approach in the extradural compartment, with a risk of opening the dura or exposing vital structures, an SP is performed. Examples include middle ear tumors, deeply located cholesteatomas (petrous bone cholesteatomas), and paraganglioas. Infection may be associated with some of these cases, reinforcing the suggestion of SP. When the dura will necessarily be opened, SP is always included. Vestibular schwannomas (or other CPA tumors) are clear examples. In both cases (extra and intradural tumors), total removal is clearly preferable if a CI is being considered.

#### 2.2.3. Inner Ear Malformations

Patients with inner ear malformations share several circumstances that make them prone to the consideration of SP. In some of them, there was a clear or reasonable probability of gusher (CSF leakage once the round window (RW) or a cochleostomy were opened). We encountered facial nerve anomalies. In many of them, promontorial access was difficult, and a posterior tympanotomy might have compromised electrode insertion. 

#### 2.2.4. Temporal Bone Transverse Fracture

Temporal bone fractures that involve the otic capsule will never consolidate. The chance of a future middle ear infection may put the patient at risk of meningitis and the need for revision surgery. In these cases, we always considered SP as the standard technique for CI. 

#### 2.2.5. Need for Good Promontorial Access

In cases of partial ossification or fibrosis of the cochlea, or large otosclerosis foci, and in cases of malformations, eliminating the middle ear provided the best exposure for electrode insertion. 

#### 2.2.6. Contracted Anatomy

There were patients with very narrow anatomy that could have enormously compromised adequate access to the RW or promontorium with conventional techniques. If, in addition, there was an inflammatory component inside the middle ear that increased bleeding, it could have hampered access to the RW. The authors always preferred an RW insertion to drilling out a cochleostomy in challenging anatomical conditions. Ménière’s disease patients were featured in the group of contracted anatomies and, especially if a concomitant labyrinthectomy was required, SP was a good option. Children may be associated with narrow anatomy and inflamed middle ear mucosa.

#### 2.2.7. Combination of Conditions

There are cases in which a combination of the abovementioned circumstances are featured. 

## 3. Results

From 2007 to 2020, a group of 92 cases in 83 patients were submitted to CI with an SP technique. All patients were managed by the senior author (M.A.). Nine patients received CI via the SP approach on both sides. Follow-up ranged from 9 months to 12.5 years, with a mean follow-up of 4.5 years. Age ranged from 11 months to 76 years, with a mean age of 28.5 years. Of the patients, 61 were male (67%) and 30 were female (33%).

Furthermore, 21 patients were in a bimodal condition, and 9 were bilateral cochlear implant users. There were five explantations, two after infection due to external trauma, and three because of poor performance.

In all but three cases, the procedure was performed in one stage. 

All cases were followed for residual disease. This was especially important for those with cholesteatoma. As MRI interferes with the area of examination, we used CT scan (both in bone window and as a cranial CT for soft tissues). Although not perfect, it allows one to identify residual disease. A CT, at a minimum of after two years, was performed in each case, with no residual disease up to now. We consider repeating the study in all cases, at an interval of two years, at least one more time. 

### 3.1. We Used an SP Approach in the Following Cases

#### 3.1.1. Chronic Otitis Media (COM)

We implanted eight patients with this condition with no postoperative infection, no re-pneumatization, and no associated complication. All patients are implant users. One of the patients with the condition after radionecrosis was staged, and performance was not optimal. Another patient with a severe inflammatory disease was staged, and it was later identified as a tuberculous (TBC) infection, which was effectively treated before CI. 

#### 3.1.2. Acute Relapsing Otitis Media

We have used this technique in four cases (three of them with associated meningitis prior to CI) with no complications. One of them required a partial drilling out of the basal turn of the cochlea. All patients are implant users with good performance. 

#### 3.1.3. Middle Ear (ME) Cholesteatoma

We used SP and CI in four cases with ME cholesteatoma. We had one case of bilateral cholesteatoma (petrous bone cholesteatoma in one side) managed with bilateral SP and binaural CI, in which the patient had demonstrated immediate postoperative autolytic behavior, contaminated both wounds, and was managed with antibiotics to try to eliminate the infection; the patient, one year later, following some revision surgeries, eventually underwent an explantation of an implant that was adequately stimulating both ears. Performance was good, and the patient could be finally reimplanted satisfactorily elsewhere. The infection came from the traumatized skin and not from the middle ear mucosa. Infection was resolved after removal of the implant, and no other complications were present. All other cases were CI users with no further complications.

#### 3.1.4. Radical Cavities

We operated on three patients with dry radical cavities, with no complications (notably no extrusions or infections), and all were regular CI users. Performance, in these cases, was not as uniform as patients with COM or even cholesteatoma.

#### 3.1.5. Petrous Bone Cholesteatoma (PBC)

We had eight cases in which an SP approach was used. Most of them required a more complex approach—the modified translabyrinthine approach (MTLB), which is associated with TLB exposure with an SP (Table 1). In one case, with preoperative long-term facial paralysis, we performed a concurrent FN rehabilitation with facial hypoglossal anastomosis. All of them worked in a bimodal condition, including one of the patients with moderate hearing loss in the contralateral side who was also operated on for middle ear cholesteatoma. One case/side was the abovementioned binaural with a PBC in one side, which required explantation after one year. There have been no other complications and no residuals so far. All seven of the other patients are implant users with a good outcome. 

#### 3.1.6. Otosclerosis 

There were four cases of otosclerosis combined with SP. One of them was associated with a radical cavity. One of the patients had a skin infection years later because of trauma and required explantation because of infection and extrusion. The patient is waiting for reimplantation. All were CI users with a good outcome. 

#### 3.1.7. Temporal Bone Transverse Fracture

There were four cases of temporal bone transverse fracture in three patients. All of them performed well with no complications. Excellent promontorial access was crucial in one case in which an extensive cochlear fibrosis required the basal turn of the cochlea to be drilled out, as it had an extensive fracture with displacement of the promontorium at the level of the basal turn. One of the patients had a bout of meningitis prior to surgery through a transverse cochlear fracture. We had no complications; all are CI users with an excellent outcome. 

#### 3.1.8. Ménière’s Disease

There were five cases of Ménière’s disease in which we used an SP approach. All of them have been used as a bimodal condition. Generally, we achieved good hearing rehabilitation that helped in spatial orientation and reduced the impact of bilateral vestibular dysfunction. Unfortunately, in one case, the CI partially failed. As the CI had a poor performance, the patient asked for the CI to be removed, and it was explanted after more than five years. No other complications were observed, and all the other patients are satisfied CI users. 

#### 3.1.9. Inner Ear Malformations

There were 30 cases in 23 patients with malformations for which we used an SP approach. Seven patients were implanted bilaterally in a sequential procedure. There were eight cases of enlarged vestibular aqueduct (EVA), five cases with a Mondini-type malformation, seven cases of incomplete partition type 3, four cases of common cavity, two cases of cochlear hypoplasia, one case of cochlear nerve hypoplasia, and three syndromic patients (Charge, Rieger, and Warsaw Breakage), many of them with difficult promontorial access. There was a re-pneumatization in one case in which the CI failed after some years and required replacement (the ET was revised and successfully closed). In another case, the electrode array entered the IAC and required immediate revision surgery, which solved the problem, repositioning the electrode array with no complications. We had to replace another CI because of failure of the device. In a severe malformed patient with Buda-type cochlear hypoplasia, blindness, mental retardation, and multiple intracranial lesions, we explanted due to doubtful performance and the need for frequent MRIs. In one case, revision surgery was necessary because of infection after some weeks; the defect was covered with temporalis fascia pedicled flap, which solved the problem effectively without the need for explantation. There were three cases of delayed facial nerve paralysis which resolved completely after weeks. Outcome in this population will depend largely on the kind of malformation. Most of the complications were due to implant failure and were not related to the SP technique. 

#### 3.1.10. Vestibular Schwannoma (VS) and Neurofibromatosis Type 2 (NF2) Patients

There were 13 cases, on 13 patients, 6 of which were NF2. We had no relevant complications. Three of the NF2 patients had null performance, although they wanted to keep the implant in case it might improve if they lose contralateral hearing. One NF2 patient asked for the CI to be removed, as he had no sound perception and requires follow-up with MRI for multiple lesions. There were 8 out of 13 patients with a good outcome. Three (the abovementioned NF2 patients) have no sound perception, but we will keep working on it. There were no serious complications in this series. In all cases, facial nerve was anatomically preserved. There were 11 patients with normal FN function, while 2 had a grade III H&B. Tumor size in these cases ran from intracanalicular to 4 cm extracanalicular. 

#### 3.1.11. Extradural Tumors

There was one case of tympanomastoid paraganglioma with bilateral hearing loss, as well as external middle ear and inner ear bilateral malformation. Furthermore, one case of geniculate ganglion lesion (later diagnosed as fibrosis) in the context of facial nerve paralysis and severe hearing loss. Both cases were implanted through an SP approach. There were no complications. Both are CI users. 

#### 3.1.12. Contracted Anatomy

We used it in seven cases with very narrow anatomy. Most of them had hypertrophy of middle ear mucosa due to chronic serous otitis media. There were no complications, and all are CI users. 

## 4. Discussion

Subtotal petrosectomy was introduced for complex temporal bone conditions and immediately became part of some complex skull base procedures. It increases surgical time, and each step should be performed meticulously to avoid potential severe complications. However, when carefully performed, it provides a safe and sterile environment, as it avoids any infectious complications arising from the eliminated middle ear mucosa, from the SRT via Eustachian tube and from the external skin. Obliteration may be performed with fat (our preferred material), musculoperiosteal flap, or bone pate [1]. The last may compromise revision surgery cases. Leaving the resultant cavity without obliteration may delay the healing process and lead to non-cosmetic deformities.

Cochlear implantation is a procedure that focuses on inner ear stimulation. In complex cases, eliminating the middle provides a safe condition, reducing the impact of infection and extrusion, preventing meningitis when CSF leaks are present, and providing the best promontorial exposure when required.

In most of the cases in our series, the procedure was performed in a single stage. We have staged in one case of middle ear tuberculosis and in one case of radionecrosis. Staging the procedure may increase safety if there is severe infection or to ensure that the tumor is not left inside. Staging the procedure could provide similar results, but in some cases, it may also increase the risk of cochlear fibrosis or ossification, making the second stage more difficult or impossible, or even worsening the performance. This is especially relevant when short-term ossification or fibrosis occur.

When compared with the posterior tympanotomy technique, the main difference for Lee et al. [11] is the higher rate of displacement of the internal device, although in complex cases, the rate of other complications compares favorably.

There are several conditions in which SP is particularly helpful.

### 4.1. Chronic Otitis Media (COM)

COM is a condition in which Eustachian tube (ET) dysfunction will remain in most of the cases. Under these circumstances, placing a cochlear implant (CI) inside the middle ear will increase the chance of implant contamination and the risk of future infection, extrusion, and explantation. SP provides a safe and sterile environment if the middle ear mucosa is eliminated, the ET is effectively closed, and the external auditory canal (EAC) is also effectively blind-sac closed. SP avoids the risk of future infections and should be strongly considered in cases of COM. Many authors have previously published on the advantages of performing an SP in cases of COM with and without discharge [1,4,5,6,7,8,9,10,12]. Preventing future middle ear infection is the most compelling reason for most authors. El Kashlan et al. [13] proposed to avoid closure of the Eustachian tube from the technique, in cases of COM, and leave a cavity connected with the rhinopharynx that can (and should) be controlled with serial imaging studies. They mentioned the advantages of avoiding fat obliteration, the possibility of detecting residuals more easily, and a simple revision surgery if required. They report on a short series of patients, with up to 7 years of follow-up, with no complications reported.

Others have proposed bypassing the middle ear in cases of COM by using the middle cranial fossa approach [14]. The potential for severe complication, the need of a different approach to the cochlea, and the use of special electrodes make it a controversial option [15].

### 4.2. Acute Relapsing Otitis Media

This is not a clear condition for SP indication associated with CI, but in cases of frequent reboots of AOM, it is worth considering [10]. The need of grommets in these cases may delay the timing of implantation or increase the chance of contamination of the implant in each bout of otitis. Getting rid of middle ear mucosa will certainly help in some of these cases. We have used this in the few cases in which meningitis previously occurred and/or AOM bouts were frequent, to avoid delayed implantation.

### 4.3. Cholesteatoma

Middle ear cholesteatoma is an unfavorable form of COM in which we expect severer ET dysfunction. The same reasons for COM are applicable here. In addition, we eliminate the option of recurrence. It is important to make sure that no residual matrix is left behind. The rate of complications is low. We had an unfortunate case in which the patient provoked a repeated autolytic contamination of the wound which ended up in a severe and long-lasting infection that required explantation. The consequences of a traumatic exposure of the implant are not directly related to the SP technique.

As with COM, many authors have proposed using this technique when combined with CI [1,4,5,6,7,8,9,10,12,16,17,18,19,20,21]. One of the differences lies in the risk of residual disease. Nevertheless, most authors accept that risk (very low) against the probability of infection. Furthermore, when fat is used as an obliterating material, HRCT may show a different density between fat and rounded residual disease, which can help in identifying it. Removal of the magnet will allow one to perform diffusion-weighted non-EPI MRI to check for residual disease, if necessary [21]. Staging the procedure may reduce persistent infection or residual disease and could be preferred by some surgeons.

### 4.4. Radical Cavities

When we have a dry cavity covered by a thin skin, it is very difficult to cover the CI without a high risk of partial exposure and extrusion. All other reasons are equal to cholesteatoma cases. Blind sac closure is more challenging, as the patient may have a large meatoplasty. This condition may be overcome by using the tragal skin as a rotating flap to match the anterior border of the skin of the resected conchal cartilage. Patients with radical cavities usually have long-term hearing loss, the inner ear epithelium may have deteriorated with time, and the results are less predictable. However, all of them are implant users. This indication is more common in some series in the literature [10]. These patients have non-draining cavities, and SP is performed to reduce the chance of extrusion as the main target together with avoiding future infections.

### 4.5. Petrous Bone Cholesteatoma (PBC)

PBC is a challenging condition and is frequently associated with bilateral chronic ear disease. In some of these cases, hearing is compromised and CI is a good option for hearing rehabilitation. Total removal is not easy and the need for imaging follow-up may be required. The presence of a CI will limit the use of MRI for this purpose, although bone window HRCT scan (as mentioned in Section 4.3) may be helpful if fat is used as material for obliteration to detect potential residuals. Diffusion-weighted non-EPI MRI may be performed, if necessary, by removing the magnet that may create a shadow impeding the vision of the surgical area [21].

In some cases, in order to reach and remove the lesion, the cochlea has to be removed, impeding the option of a CI. However, when the cochlea may be partially or completely preserved, and especially if there is residual hearing, CI is a good option. In most of the cases, SP is an essential part of the technique to avoid complications due to exposure of the dura, sigmoid sinus, jugular bulb, and internal carotid artery and to protect from a potential leakage through the dura or the IAC. When a CSF leak is present, SP is even more essential. All of them have been used in a bimodal condition with a contralateral, compromised or at-risk of hearing loss, because of COM or cholesteatoma, and they are highly satisfied implant users.

PBC cases should be considered to be at high risk of deep residual disease, and each case should be carefully evaluated before considering CI. If there is a high risk of residual disease and a clear indication for CI, the implantation could be staged, leaving a dummy electrode, as the risk of cochlear fibrosis after translabyrinthine exposure of the IAC is high.

CI, in cases of petrous bone cholesteatoma, has not been mentioned before in the literature.

### 4.6. Otosclerosis

Otosclerosis, per se, is not a clear indication for SP in cases of CI, but when there is a RW or extensive basal turn involvement by otospongiotic bone in cases of very advanced otosclerosis, especially if we have a narrow anatomy, CI with SP is a good option for excellent promontorial access. In one case, drilling out of the cochlea was extensive and certainly facilitated by removal of the EAC. Another case was associated with a radical cavity on an overlapping indication for SP. There was also a case of explantation because of external trauma and infection which could not be controlled with conservative management.

In the literature, some of these cases are referred to as cochlear ossification [8,10,21].

### 4.7. Temporal Bone Transverse Fracture

Eliminating the middle ear in cases of a transverse fracture that will never consolidate prevents meningitis from future potential middle ear infections, as was the case in one of our patients prior to implantation. We strongly considered and advised combining SP with CI in these cases. This condition has been included in the literature [8,10].

### 4.8. Ménière’s Disease

Ménière’s disease is not a routine indication for SP for us, but Ménière’s disease patients usually have a contracted anatomy with a narrow Trautman’s triangle. When a labyrinthectomy has to be associated with a contracted mastoid, as was the case in all our patients, SP is preferred. Furthermore, when intratympanic therapy has been used with unsuccessful results, and a drainage tube is present with associated otorrhea, eliminating the middle ear allows prevention of future middle ear infections; a labyrinthectomy provides an excellent outcome in terms of vertigo relief, and CI allows hearing rehabilitation in these patients. In doing so, we can achieve three goals in succession. These patients clearly benefit from hearing rehabilitation for their vestibular rehabilitation as they improve spatial orientation, especially in cases of bilateral Ménière’s disease. SP helps in creating a safe condition for these handicapped patients.

### 4.9. Inner Ear Malformations

Inner ear malformations comprise a group of very different scenarios. In some of them, we cannot anticipate an excellent outcome like severe cochlear hypoplasia or common cavities. In others, we can expect a good outcome, as in enlarged vestibular aqueduct (EVA), but there are two issues that make malformations challenging: a potential intraoperative gusher and a malformed facial nerve [22]. SP offers the best approach to overcome these limitations and offers excellent promontorial access. Gusher may be anticipated in cases of EVA, incomplete partition type I and III (cochlea with absent modiolus), common cavities, and in some cases of Mondini malformation (which also associates with EVA). EVA patients will present more commonly with discharge coming from the perilymphatic space that would also increase the chance of meningitis if middle ear infection occurs. Some will have a more important gusher coming from the subarachnoid space. Closure of the ET prevents future middle ear infections (common in children) and the occurrence of meningitis. When the cochlea is abnormal, it benefits from the best promontorial access provided by the SP approach. Some syndromic patients, such as those with CHARGE syndrome, are very challenging [23]. This malformed population are complex cases, very variable in outcome, and include one the highest rates of complications in this group of SP-CI patients. Nevertheless, many were related to a delayed failure of the device and not to the technique. Extensive drilling in children close to the third portion of the facial nerve, as required, increases the chance of delayed facial nerve paralysis, which will completely recover in the short term, as was the case in all three of our patients.

### 4.10. Vestibular Schwannoma (VS) and Neurofibromatosis Type 2 (NF2) Patients

Traditionally, auditory brain stem implant (ABI) was considered the standard option to rehabilitate patients with VS, especially NF2 patients. When preserving the cochlear nerve, it soon became evident that a CI would provide a better outcome. Patients submitted for surgery for VS and/or NF2 with poor or absent residual hearing or bad candidates for hearing preservation may be considered for CI. Simultaneous CI provides the best option for these patients. Normal hearing, good hearing, or even poor residual hearing are good indicators for a chance of cochlear nerve preservation. On the other hand, patients with no residual hearing may be poor candidates. Intraoperative cochlear nerve monitoring may help in decision making. An intraoperative test electrode (Auditory Nerve Test Electrode—ANTE) has been developed and tested in a multicenter study with 93% accuracy [24]. Electrode insertion may be performed through a classic posterior tympanotomy approach, but the chance of rhinoliquorrhea complicating the procedure and forcing a reintervention with the CI in place is a strong reason for us to consider eliminating the middle ear. As already stated, associating SP with a translabyrinthine approach (modified translabyrinthine approach—MTLB) may reduce the rate of CSF leak [25]. At the same time, it provides excellent promontorial access and allows for a good stabilization of the guide of the electrodes. We have used this modified approach, which includes SP, in all cases of VS and NF2 in which we have placed a CI, and some of this series has been previously reported in the literature [26]. NF2 patients are less predictable in terms of outcome and include all the non-implant users, and they were the only ones who required explantation because of poor performance and the need for MRI follow-up. MRI, nevertheless, does not impede follow-up of the IAC and CPA searching for residual disease. We have added this group of patients to the series because all the steps of the SP are included in the technique, and they share with any other group of patients considered the potential risks of eliminating the middle ear.

### 4.11. Extradural Tumors

There was one case of tympanomastoid paraganglioma with bilateral hearing loss and external middle ear and inner ear malformation. A challenging case in which CI was staged to deal with potential residual.

There was also one case of geniculate ganglion lesion (later diagnosed as fibrosis) and severe hearing loss with partial fibrosis of the basal turn of the cochlea.

Both cases were implanted using an SP approach. There were no complications. Both are CI users.

In case of intratemporal tumors, follow-up for potential residuals with MRI may require removal of the magnet. Octreotide, in case of paragangliomas, and staging in other cases, may help to overcome this situation. This condition is mentioned in the literature [27].

### 4.12. Contracted Anatomy

When CT scan anticipates a very narrow anatomy, SP is a technique worth considering to avoid difficulties during surgery and provides excellent promontorial access. This has been the case in children with swollen mucosa (chronic serous otitis media) and a contracted mastoid. This indication may be controversial, but it has the advantage of preventing the impact of potential bouts of acute otitis media in patients with a cochlear implant in site. Other authors have also included this condition as an indication of CI associated with SP [8,10,21,27].

### 4.13. Meta-Analysis

Yan et al. recently published a meta-analysis on the topic of SP and CI [27]. They report on 377 patients (394 SP) from 27 studies. The largest series (110 cases) comes from a multicenter study. In this revision, 24.1% required multistage procedures compared with 2.1% in our series. In cases of cholesteatoma, 42.6% underwent a multistage procedure compared with 0% in our series. The overall complication rate was 12.4% (9.4–15.9%) compared with 13% in the present series. Most of the major complications in our cases were due to delayed CI failures or trauma and infection, which led to explantations.

The study did not find differences in complication rates across age groups. In our series, the largest group (30) are inner ear malformations, mainly in children, which include the highest rate of complications.

Indications included COM in 55.4% (8.7% in the present series—PS), radical cavity in 35.5% (3.3% PS), cholesteatoma in 18.6% (13% PS), inner ear malformations in 4.0% (32.6% PS), temporal bone fracture in 3.8% (4.3% PS), and unfavorable anatomy in 4.3% (7.6% PS). Other indications in the series included recurrent acute otitis media and cochlear ossification. We have included, in our series, patients with very advanced otosclerosis, Ménière’s disease, and vestibular schwannoma (in which we always associate with an SP). Our series, then, has a different distribution when compared with the current literature, making comparison of overall complication rates inconclusive.

Cholesteatoma recidivism rate was 9.3% (0% in the present series), reoperation rate was 10.2% (9.7% PS), and device failure was 7.3% (3.26% PS). There were five facial palsies (one permanent and four transient) compared to three (all transient) in our series.

In the present series, a high number of children with inner ear malformation and NF2 cases include most of the complications.

SP compares favorably with complications reported by Hunter et al. in a systematic review and meta-analysis of the literature after canal wall down (CWD) mastoidectomy [28] with a complication rate of 30%.

## 5. Conclusions

Subtotal petrosectomy is an accurate, and sometimes difficult, technique that increases surgical time, but it provides a safe and sterile environment, and excellent promontorial access for complex cases of cochlear implantation for all ages. Among these indications are chronic ear disease (including middle ear cholesteatoma and petrous bone cholesteatoma), inner ear malformations, very advanced otosclerosis, temporal bone fractures, Ménière’s disease, vestibular schwannoma, and other tumors. The rate of complications compares favorably with standard techniques. SP should be strongly considered when approaching cases of cochlear implantation with unfavorable conditions.

## Figures and Tables

**Table 1:** Distribution of patients by indication. SP, subtotal petrosectomy; MTLB, modified translabyrinthine approach; LAB, labyrinthectomy; DFN, delayed facial nerve.

Indication	No. of Cases Approach	CI Users	Complications
Chronic otitis media	8 SP (2 staged)	8	None
Acute relapsing otitis media	4 SP (1 drill out)	4	None
Cholesteatoma	5 SP (1 binaural 1 side Lab)	4	1 explantation (post-trauma skin infection)
Petrous bone cholesteatoma	7 7 MTLB	7	Skin infection without extrusion
Radical cavity	3 SP	3	None
Otosclerosis	4 SP	3	1 trauma > infection after 5 years with explantation
TB transverse fracture	4 SP	4	None
Ménière’s disease	5 4 LAB + SP 1LAB > MTLB VN	4	1 Explantation (poor performance)
Inner ear malformations	30 SP	29	1 explantation (Poor P) 2 Replacements 3 DFN paralysis 1 skin infection
Vestibular schwannoma (6 NF2)	13 13 SP + TLB (MTLB)	11	1 Explantation poor performance (NF2) 1 non-user null perform (Nf2) 2 users null performance (NF2)
Extradural tumors	2 SP	2	None
Contracted anatomy	7 SP	7	None
Total	92 SP	86	12 (13%)

## Data Availability

Data supporting reported results can be found at the guarded Data Base of the Senior Author in the Hospital under key code access.
